# Anti‐Inflammatory Role of Myo‐Inositol in Obesity: Suppression of TNF‐α‐Induced Inflammation and Monocyte Adhesion in Hypertrophic Human Adipocytes

**DOI:** 10.1002/fsn3.70962

**Published:** 2025-09-12

**Authors:** Stefano Quarta, Nadia Calabriso, Maria Annunziata Carluccio, Marta Lo Rizzo, Martin Wabitsch, Tiziano Verri, Michele Maffia, Fabrizio Damiano, Luisa Siculella, Giuseppe Santarpino, Marika Massaro

**Affiliations:** ^1^ Institute of Clinical Physiology (IFC) National Research Council (CNR) Lecce Italy; ^2^ Department of Biological and Environmental Sciences and Technologies (DiSTeBA) University of Salento Lecce Italy; ^3^ Division of Pediatric Endocrinology, Diabetes and Obesity, Department of Pediatrics and Adolescent Medicine, German Center for Child and Adolescent Health (DZKJ), Partner Site Ulm University of Ulm Ulm Germany; ^4^ Department of Experimental Medicine (DiMeS) University of Salento Lecce Italy; ^5^ Cardiovascular Center Paracelsus Medical University Nuremberg Germany; ^6^ GVM Care & Research Città di Lecce Hospital Lecce Italy; ^7^ Cardiac Surgery Unit, Department of Experimental and Clinical Medicine University “Magna Graecia” Catanzaro Italy

**Keywords:** adipocytes, adipose‐monocyte interaction, inflammation, inositols, NF‐κB

## Abstract

Inflammation in hypertrophic adipose tissue is a key driver of obesity‐related cardiometabolic diseases. Under insulin resistance conditions, insulin signaling shifts toward pro‐inflammatory pathways, disrupting normal metabolic processes. Inositols (INSs), a class of carbocyclic sugars, serve as important secondary messengers in insulin signaling. This study investigates whether myo‐inositol (MYO), in addition to its known insulin‐sensitizing properties, also exerts anti‐inflammatory effects, and thus potentially attenuates adipose tissue inflammation. Human Simpson‐Golabi‐Behmel syndrome (SGBS) adipocytes were treated with MYO (100 μmol/L) for 4 h before pro‐inflammatory stimulation with different dymetabolic‐related cytokines including tumor necrosis factor (TNF)‐α, interleukin (IL)‐1β, and lipopolysaccharide (LPS). Pro‐inflammatory gene expression and protein secretion were assessed via qPCR, ELISA, EIA, and immunocytochemistry, while reactive oxygen species (ROS) overproduction, nuclear factor (NF)‐κB activation, and mitochondrial content were assessed using specific probes and transactivation assays, respectively. Functionally, the regulation of adhesion of monocytes to inflamed adipocytes was quantified using a cell adhesion assay. MYO significantly reduced the pro‐inflammatory expression and secretion of CCL‐2, CXCL‐10, and IL‐6, resulting in reduced adhesion of monocytes to inflamed adipocytes. Additionally, MYO suppressed TNF‐α‐induced surface expression of ICAM‐1, a critical adhesion molecule in adipose tissue inflammation. Mechanistically, MYO attenuated ROS overproduction and the related NF‐κB activation, the key regulator of adipocyte inflammation, and improved the mitochondrial dysfunction, thus overall restoring adipose tissue dysfunction. Although further research is needed, these findings suggest that MYO effectively improves the inflammatory and dysmetabolic profile of hypertrophic fat cells, highlighting its potential as a therapeutic option for metabolic disorders.

## Graphical abstract

Supplements targeting adipose tissue inflammation may reduce obesity‐related cardiometabolic diseases. We tested whether Myo‐Inositol (MYO), a carbocyclic sugar with insulin‐sensitizing effects, reduces adipose tissue inflammation using an in vitro model of human adipocytes stimulated with TNF‐α. Reducing the TNF‐α‐induced NF‐κB activation, MYO mitigates the expression and release of cyto‐chemokines and adhesion proteins, as well as monocytes adhesion and migration towards activated adipocytes. Although with some limitations, these data suggest that MYO improves the inflammatory and dysmetabolic milieu of hypertrophic adipocytes.

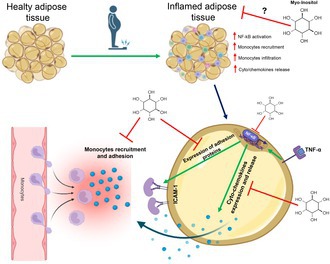

**Abbreviations**
CXCL‐10: C‐X‐C motif chemokine ligand‐10
DCI: D‐chiro‐inositol
ICAM‐1: intercellular adhesion molecule‐1
IL‐6: interleukin‐6
MCP‐1: monocyte chemoattractant protein‐1
MYO: myoinositol
NF‐κB: nuclear factor‐κB
TNF‐α: tumor necrosis factor‐α

## 1 Introduction

In individuals with severe obesity or those who have exceeded their physiological capacity to store fat, a combination of systemic low‐grade inflammation and intense localized inflammation in adipose tissue plays a pivotal role in the development of metabolic disorders, including insulin resistance (IR) and type 2 diabetes (T2D) (Wu and Ballantyne 2020). Although the precise triggers of adipose tissue inflammation and IR in obesity remain unclear, several contributing factors have been identified. One key factor is the mechanical stress induced by excessive enlargement and subsequent shrinkage of hypertrophic fat cells. Enlarged adipocytes experience heightened endoplasmic reticulum (ER) stress and generate excessive reactive oxygen species (ROS), which activate multiple intracellular signaling pathways, leading to the release of pro‐inflammatory cytokines (Zhou et al. 2021). This dysregulated cytokine expression is characterized by increased levels of tumor necrosis factor‐α (TNF‐α), interleukin‐6 (IL‐6), and monocyte chemoattractant protein‐1 (MCP‐1), alongside reduced levels of adiponectin (Zatterale et al. 2019). Compared to lean individuals, obese individuals have significantly higher TNF‐α expression in adipose tissue (Quarta, Massaro, et al. 2022), which correlates with hyperinsulinemia (Hotamisligil et al. 1995). TNF‐α impairs insulin signaling by inhibiting the phosphorylation of insulin receptor substrate (IRS)‐1 and the activation of phosphatidylinositol 3‐kinase (PI3K)/protein kinase Akt through the activation of Mitogen‐Activated Protein Kinases (MAPKs) and IκB kinase (IKK) (de Alvaro et al. 2004; Fujishiro et al. 2003). This prevents effective insulin signaling transduction (Ishizuka et al. 2007) while promoting transcriptional pro‐inflammatory activity of nuclear factor‐κB (NF‐κB) (Zatterale et al. 2019). These inflammatory changes lead to the recruitment of pro‐inflammatory leukocytes, which secrete additional TNF‐α and amplify the inflammatory cascade, further exacerbating the dysmetabolic state (Boutens et al. 2018).

Given the central role of inflammation in obesity, targeting inflammation has emerged as a promising therapeutic strategy for obesity‐related diseases (Wu and Ballantyne 2020). However, in clinical practice, treating metabolic diseases through inflammation‐targeting therapies remains challenging and often yields unsatisfactory results (Goldfine and Shoelson 2017). Obesity and its related inflammatory conditions are chronic, long‐term processes, and while anti‐inflammatory treatments offer potential benefits, their long‐term systemic side effects often pose serious health dangers (Wu and Ballantyne 2020). As a result, adopting less invasive strategies, such as nutritional supplements, could serve as a valuable complementary approach, providing therapeutic advantages with a reduced risk of adverse effects.

Inositols (INSs) are carbocyclic sugar polyalcohols that occur in several stereoisomeric forms (Bizzarri et al. 2016). Myo‐Inositol (MYO), the most abundant isomer, is synthesized endogenously from D‐glucose and converted to D‐Chiro‐Inositol (DCI) by an NADH‐dependent epimerase reaction (Pani et al. 2020). In addition, INSs can be obtained from various food sources, with particularly high concentrations in fruits, beans, cereals, and nuts (R. Clements and Darnell 1980). MYO and its derivatives are endowed with insulin‐mimetic activity (Sánchez‐Hidalgo et al. 2021). They play a role as a secondary messenger in insulin signaling and are known to regulate insulin secretion, mitochondrial function, and glycogen storage (Sánchez‐Hidalgo et al. 2021). Moreover, INSs have shown significant antioxidant and anti‐inflammatory effects in various pathological conditions (Sánchez‐Hidalgo et al. 2021), and specifically affect the activation of PI3K/Akt (Cheng et al. 2019). Considering these findings, we were prompted to investigate the ability of MYO to interfere with TNF‐α‐induced pro‐inflammatory dysregulation of adipose tissue gene expression and its interaction with monocytes, while also exploring the underlying mechanisms of action.

## 2 Materials and Methods

### 2.1 Materials

Myo‐Inositol (MYO) is a component of NITRONAT Plus and was provided by Essecore S.R.L. (Essecore, Bari, Italy). Chemicals and reagents used to measure mRNA levels were obtained from Bio‐Rad Laboratory (Hercules, CA, USA). Unless otherwise indicated, all the other chemicals were from Sigma Aldrich (St. Louis, MO, USA).

### 2.2 Cell Culture and Treatments

Simpson‐Golabi‐Behmel syndrome (SGBS) preadipocytes were maintained in culture and differentiated into adipocytes, as described in previous research (Massaro et al. 2016; Wabitsch et al. 2001). Differentiated adipocytes were exposed to MYO for up to 72 h and then stimulated with 10 ng/mL TNF‐α for 1 to 24 h. THP‐1 cells, as a model of human monocytic cells, were purchased from the American Tissue Culture Collection (Rockville, MD, USA) and cultured as described in previous research (Quarta, Santarpino, et al. 2022).

### 2.3 Cell Viability

Cell viability was assessed using the MTT (3‐(4,5‐dimethylthiazol‐2‐yl)‐2,5‐diphenyltetrazolium bromide) assay, which measures the ability of viable cells to reduce MTT into an insoluble formazan product that can be quantified spectrophotometrically. Briefly, after treatment with Myo‐Inositol and exposure to TNF‐α, cells were incubated with MTT (0.5 mg/mL) for 3 h. The resulting formazan crystals were dissolved in isopropanol, and absorbance was measured at 490 nm using a microplate reader.

### 2.4 Assessment of Cellular Lipid Accumulation

Intracellular lipid accumulation was assessed using Oil Red O (ORO) staining, following the protocol outlined by Quarta et al. (2021). After MYO treatment, cells were rinsed twice, fixed in 10% formalin for 30 min, and subsequently stained with ORO solution for 1 h. To eliminate unbound dye, cells were washed twice before imaging with an inverted microscope (Leica, Wetzlar, Germany). Following image acquisition, the retained ORO stain was extracted using isopropanol, and absorbance was measured at 510 nm using a microplate reader. The OD510 value was considered a surrogate marker for intracellular triglyceride accumulation.

### 2.5 RNA Isolation and Real‐Time Quantitative Polymerase Chain Reaction

Total RNA was extracted, reverse‐transcribed into cDNA, and assayed by quantitative real‐time PCR (qPCR) as previously described (Quarta, Santarpino, et al. 2022). The primer sequences used are detailed in Table 1.

**TABLE 1:** Primer sequences.

Gene symbol	Full name	Forward primer	Revers primer	Accession number
MCP‐1	Monocyte chemoattractant protein‐1	CCCCAGTCACCTGCTGTTAT	TCCTGAACCCACTTCTGCTT	NM_002982.3
IL‐6	Interleukin‐6	AGGAGACTTGCCTGGTGAAA	CAGGGGTGGTTATTGCATCT	NM_000600.5
CXCL‐10	C‐X‐C Motif chemokine ligand‐10	CAAGGATGGACCACACAGAG	GCAGGGTCAGAACATCCACT	NM_001565.2
ICAM‐1	Intercellular adhesion molecule‐1	AGACATAGCCCCACCATGAG	CAAGGGTTGGGGTCAGTAGA	NM_000201.2
18S	18 ribosomal RNA	AAACGGCTACCACATCCAAG	CCTCCAATGGATCCTCGTTA	NR_003286.2

### 2.6 Measure of Chemokines Release

The conditioned media from SGBS were collected under sterile conditions, centrifuged to remove cell debris, and frozen at −20°C until use. The levels of secreted MCP‐1, IL‐6, and CXCL‐10 were determined using the corresponding enzyme‐linked immunosorbent assay (ELISA) kit, according to the manufacturer's instructions.

### 2.7 In Vitro THP‐1 Chemotaxis Assay

MYO‐conditioned SGBS media, previously used for chemokine release measurements, were also utilized to evaluate THP‐1 migration using a Boyden chamber as previously described (Quarta, Santarpino, et al. 2022). Briefly, THP‐1 cells were placed in the upper chamber, while the SGBS‐conditioned medium was added to the lower chamber. Following a 60‐min incubation at 37°C, the upper chamber was removed, and migrated THP‐1 cells were quantified by adding MTT. The role of MCP‐1 in monocyte migration was assessed by adding a recombinant MCP‐1 to the upper chamber. This significantly inhibited monocyte migration by disrupting the chemoattraction gradient.

To assess the role of chemoattractants such as MCP‐1 in monocyte migration, a recombinant MCP‐1 solution was introduced into the upper chamber. This significantly reduced monocyte migration by disrupting the chemoattraction gradient, confirming MCP‐1's involvement in the process.

### 2.8 Cell Surface Immunoassay

Surface expression of cell adhesion molecules on SGBS was measured using a surface enzyme immunoassay (EIA) as previously described (Massaro et al. 2002).

### 2.9 Immunocytochemistry

SGBS were differentiated in 24‐well plates and incubated with MYO for 4 h and then stimulated with 10 ng/mL TNF‐α for 16 h. After removal of culture supernatants, monolayers were fixed with 1% paraformaldehyde. Then, nonspecific binding sites were blocked with 0.1% FBS for 30 min. Cells were then exposed to mouse monoclonal antibodies against ICAM‐1 for 2 h at 4°C before washing with PBS/0.1% FBS and incubation with a fluorescence goat anti‐mouse IgG (Thermo Fisher Scientific, Waltham, Massachusetts, USA) for 1 h. Cells were then washed thoroughly and visualized and captured with an EVOS Floid fluorescence microscope (Thermo Fisher Scientific).

### 2.10 In Vitro THP‐1 Adhesion Assay

To determine the number of monocytes adhering to the cultured adipocytes, THP‐1 cells were fluorescently labeled with calcein‐AM for 30 min. Labeled THP‐1 cells were then added to the SGBS monolayers. After 1 h, non‐adherent cells were removed by gently washing, and the monolayers were fixed with 1% paraformaldehyde. Images of the SGBS monolayers with adherent calcein‐labeled THP‐1 cells were captured using a stereomicroscope (Nikon, Minato, Tokyo, Japan) with Nikon NIS‐Elements software at 40× magnification.

### 2.11 Preparation of Nuclear Extracts and Measurement of NF‐κB p65 DNA Binding Activity

Nuclear proteins were obtained using the Nuclear Extract kit (Active Motif, Carlsbad, CA, USA), in accordance with the manufacturer's instructions. The activation of NF‐κB was measured using the ELISA‐based TransAM NF‐κB p65 kit (Active Motif, Carlsbad, CA, USA), following the protocol provided by the manufacturer.

### 2.12 Measuring ROS Levels

To investigate the impact of MYO on ROS production in differentiated adipocytes, SGBS cells were seeded in 96‐well plates, induced to differentiate, and subsequently treated with MYO for 4 h. Following treatment, cells were stimulated with 10 ng/mL TNF‐α for 30 min. After stimulation, cells were washed twice and incubated with 40 μM 2′,7′‐Dichlorofluorescin Diacetate (DCF‐DA, Invitrogen, Thermo Fisher Scientific) in PBS at 37°C for 30 min. Fluorescence intensity was then measured using a microplate reader with excitation at 485 nm and emission at 535 nm to quantify ROS levels.

### 2.13 Mitochondrial Content Measurement

The mitochondrial content was determined using the Mitotracker red cmxros probe (Thermo Fisher Scientific). After treatment with MYO for 4 h and stimulation with TNF‐α for 16 h, the cells were incubated with 200 nmol/L of the probe in serum‐free medium at 37°C for 30 min and fluorescence intensity was measured using a microplate reader at excitation and emission wavelengths of 644 and 665 nm, respectively. The cells were then fixed with 4% paraformaldehyde for 10 min and permeabilized with 0.1% Triton X‐100 in PBS for 10 min. The cell nuclei were stained with 4′,6‐diamidino‐2‐phenyl‐indole (DAPI, Roche Diagnostics, Indianapolis, IN, USA) for a further 10 min at room temperature. After a final wash with PBS, images of the cells were captured under an EVOS Floid fluorescence microscope.

### 2.14 Statistical Analysis

Results are expressed as means ± SD of at least three independent experiments performed in triplicate. Student's *t* test was used for comparison of means between the control group and compound‐treated group. A *p* level of < 0.05 was considered statistically significant.

## 3 Results

### 3.1 Effect of Myo‐Inositol on Adipocyte Cell Viability and Lipid Content

**FIGURE 1:**
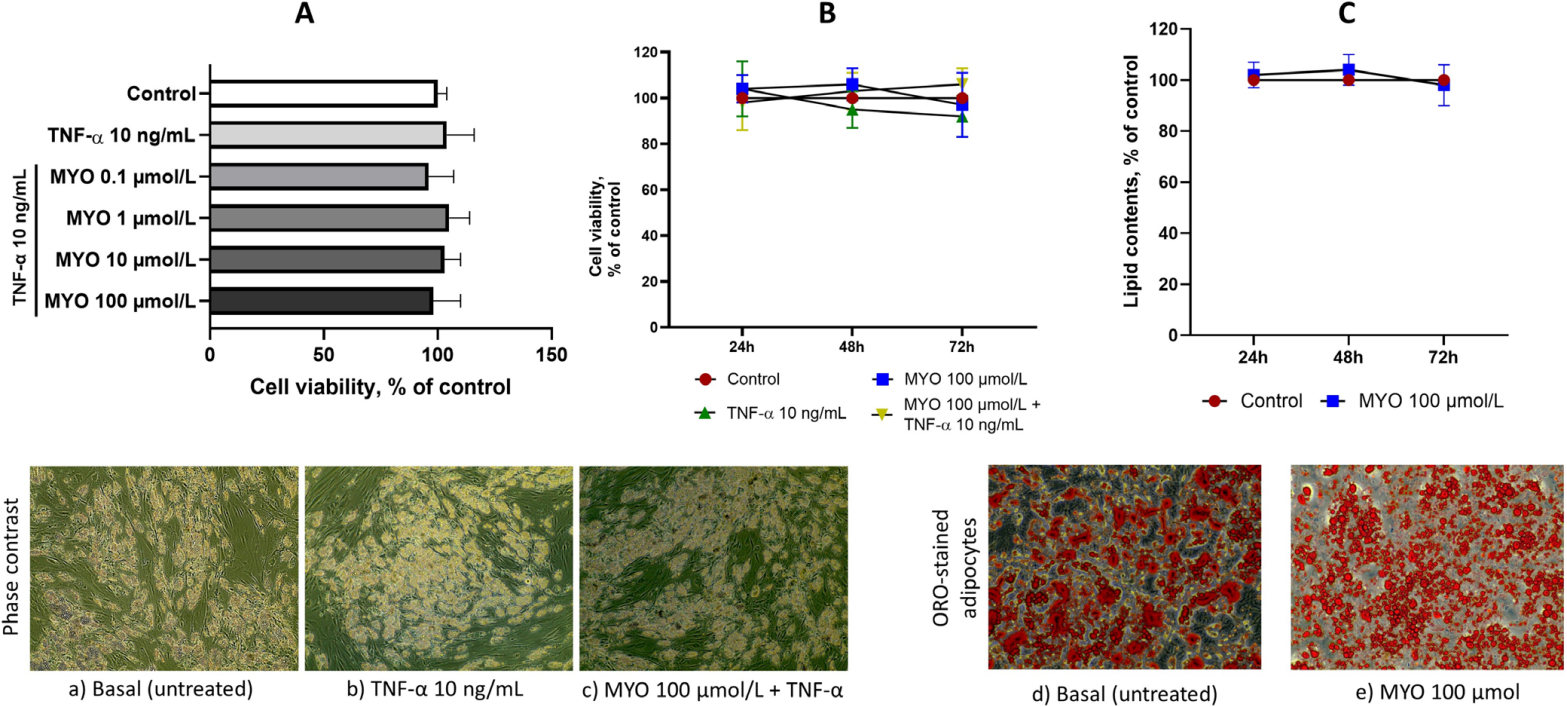
Effect of MYO on cell viability and lipid accumulation. (A) Adipocytes were treated with MYO for up 4 h at the concentrations indicated, and then either treated with 10 ng/mL TNF‐α or left untreated for 16 h. Cell viability was assessed by a 3‐(4,5‐dimethylthiazol‐2‐yl)‐2,5‐diphenyl tetrazolium bromide (MTT) assay, and expressed as a percentage of the unstimulated control. Data (means ± SD, *n* = 3). In the lower panels (a–c) SGBS representative images were acquired with a phase contrast microscope at 10× magnification. (B) Adipocytes were treated with MYO for up 72 h, and then either treated with 10 ng/mL TNF‐α or left untreated for 16 h. Cell viability was assessed by a 3‐(4,5‐dimethylthiazol‐2‐yl)‐2,5‐diphenyl tetrazolium bromide (MTT) assay, and expressed as a percentage of the unstimulated control. Data (means ± SD, *n* = 3). (C) Adpocytes were treated with MYO for 24 h. Intracellular lipid accumulation was evaluated by Oil Red O (ORO) staining. In the upper panel it is reported the spectrophotometric quantification of ORO stain extracted from SGBS adipocytes. Data (means ± SD, *n* = 3) are expressed as percentage over basal (untreated) control. In the lower panels (d, e) are reported the microphotographs of ORO stained SGBS adipocytes in the absence (a) or in the presence (b) of 100 μmol/L MYO.

Preliminary experiments were conducted to test the effects of MYO on cell viability and lipid content. Based on a previous pharmacokinetic study showing an increase in plasma MYO concentration from 30 to 120 μmol/L after an oral dose of 3.0 g MYO in both healthy and diabetic subjects (R. S. Clements Jr. and Reynertson 1977), fully differentiated SGBS adipocytes were pretreated with MYO at a concentration range of 0.1 to 100 μmol/L for up to 72 h and then stimulated with 10 ng/mL TNF‐α for up to 72 h. After the treatments, cell viability was determined using the MTT assay. As shown in Figure 1A,B, MYO had no significant effect on cell viability, even at higher concentrations, either in the absence or presence of TNF‐α. No change in cell morphology was observed (Figure 1a–c). Therefore, we used 100 μmol/L as the standard concentration of MYO for all further experiments.

At this concentration, treatment of adipocytes with MYO for up to 72 h had no effect on triglyceride storage, as shown by spectrophotometric evaluation of lipid content (Figure 1C) and representative ORO‐stained cell images (Figure 1d,e).

### 3.2 MYO Reduces Inflammatory Gene Expression and Monocyte Chemotaxis Induced by Various Pro‐Inflammatory Stimuli

**FIGURE 2:**
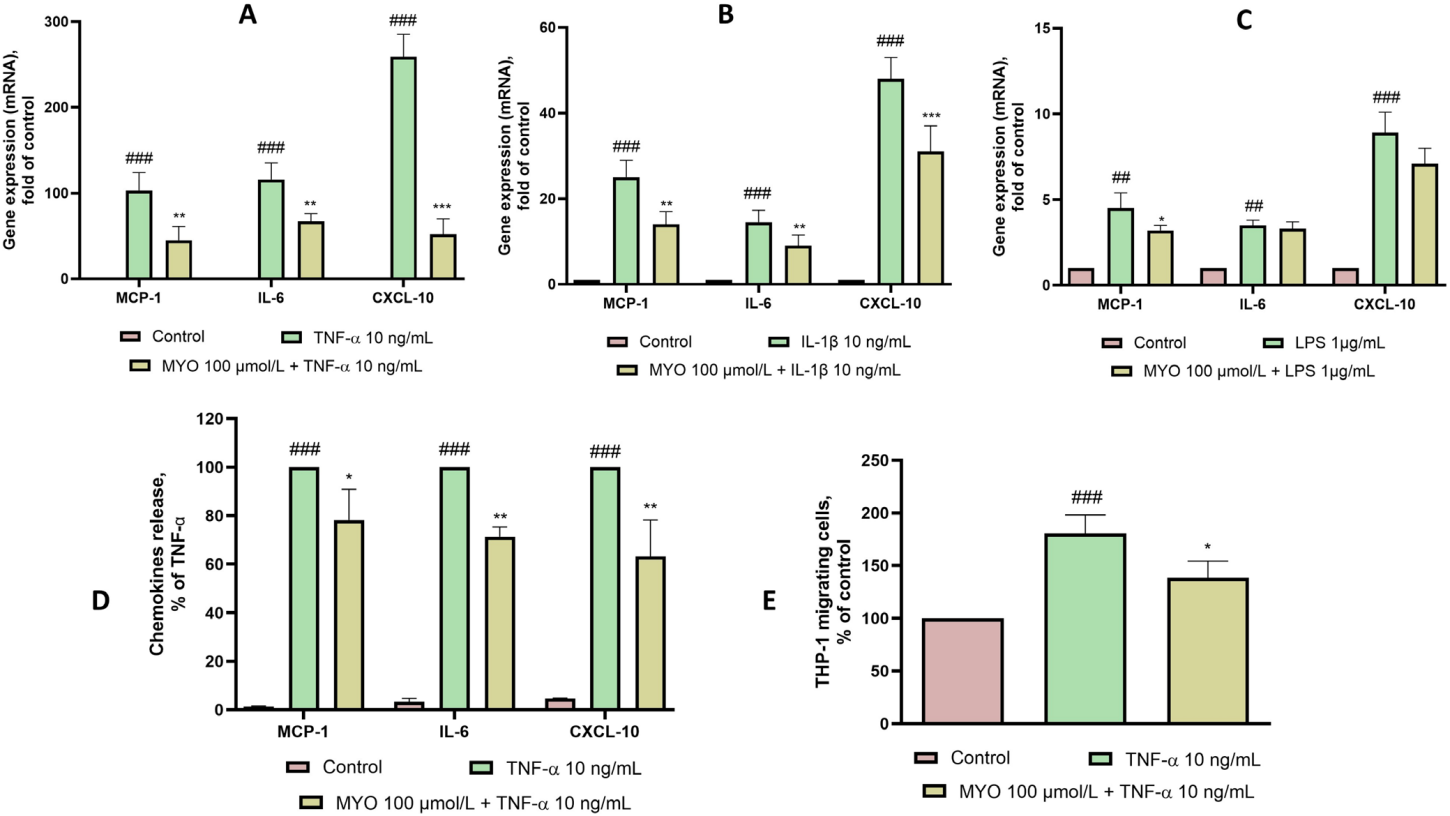
MYO reduces pro‐inflammatory‐induced chemokines gene and protein expression. Adipocytes were treated with MYO for 4 and then either treated with 10 ng/mL TNF‐α (A), IL‐1β (B), and LPS (C) or left untreated for 16 h. Culture medium was collected under sterile conditions. Total RNA was extracted from cells, and mRNA levels of MCP‐1, IL‐6, CXCL‐10 were measured by qPCR using specific primers and normalized to 18S rRNA. Data (means ± SD, *n* = 3) are expressed as fold induction over basal (untreated) control. ^###^ *p* < 0.001 challengers versus basal (untreated) control; ^##^ *p* < 0.05 challengers versus basal (untreated) control **p < 0.01 MYO versus challengers; ***p < 0.001 MYO versus challengers. (D) MCP‐1, IL‐6, and CXCL‐10 protein release was evaluated by ELISA assay. Data (mean ± SD, *n* = 3) are expressed as % versus TNF‐α. ^###^ *p* < 0.001 versus basal (untreated) control; *p < 0.05 versus TNF‐α alone; **p < 0.01 versus TNF‐α alone. (E) Culture medium was added to the lower chamber in a Boyden chamber. THP‐1 (2.5 × 10^6^ cells/mL) were added to the upper chamber. After 60 min, migrated THP‐1 cells were measured by MTT assay. Data (means ± SD, *n* = 3) are expressed as % of migrated monocytes over control. ^###^ *p* < 0.001 versus basal (untreated) control; *p < 0.05 versus TNF‐α alone.

Adipocyte activation and dysfunction are characterized by an increased expression of inflammatory cytokines and chemokines that drive the recruitment of monocytes to the hypertrophic adipose tissue (Zatterale et al. 2019). We investigated the effects of MYO on stimulated mRNA expression and the release of several chemokines, including MCP‐1 (CCL‐2), IL‐6, and CXCL‐10, induced by several challengers relevant to adipose dysfunction, including TNF‐α (Sethi and Hotamisligil 2021), IL‐1β (Ghanbari et al. 2021) and the recently recognized gut microbiota‐derived lipopolysaccharide (LPS) (Hersoug et al. 2018). As shown in Figure 2A,B, both TNF‐α and IL‐1β highly induced the expression of MCP‐1, IL‐6, and CXCL10, which were effectively reduced by MYO. In comparison, LPS induced a milder pro‐inflammatory response, and the inhibitory effect of MYO was more limited, significantly reducing only MCP‐1 levels (Figure 2C). We further investigated the ability of MYO to inhibit protein release of these chemokines and cytokines. As shown in Figure 2D, MYO effectively reduced protein secretion of all mediators tested, with the release of MCP‐1 decreasing by approximately 20% (*p* < 0.05), IL‐6 by approximately 30%, and CXCL10 by almost 40% (*p* < 0.01). From a functional point of view, these inhibitory effects by MYO on chemokine release in the inflamed adipocytes agreed with the observation of a significant decrease in monocyte chemotaxis toward conditioned media of SGBS pretreated with MYO compared with TNF‐α alone (Figure 2E).

### 3.3 MYO Reduces ICAM‐1 Expression and Monocyte Adhesion on Inflamed Adipocytes

After observing that MYO reduces the stimulated release of chemotactic cytokines, an effect functionally confirmed by the decreased ability of adipose supernatant to attract monocytes, we took the next step to assess whether MYO could also directly inhibit the interaction between inflamed adipose cells and monocytes.

**FIGURE 3:**
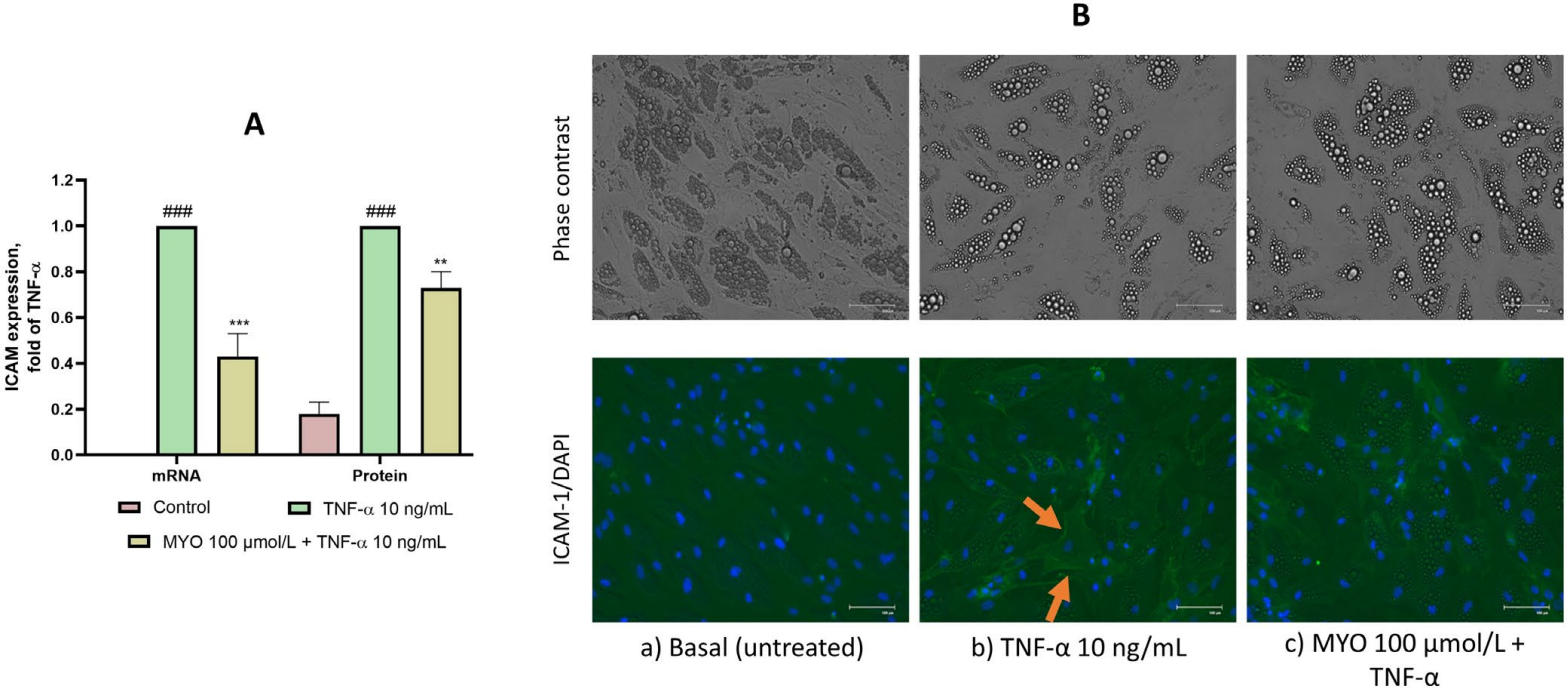
MYO reduces ICAM‐1 expression in inflamed adipocytes. Adipocytes were treated with MYO for 4 h and then either treated with 10 ng/mL TNF‐α or left untreated for 16 h. (A) Total RNA was extracted from cells, and mRNA levels of ICAM‐1 were measured by qPCR using specific primers and normalized to 18S RNA. Cell surface expression of ICAM‐1 was assessed by cell surface EIA. Data (means ± SD, *n* = 3) are expressed as fold induction over TNF‐α. ^###^ *p* < 0.001 versus basal (untreated) control; **p < 0.01 versus TNF‐α alone; ***p < 0.001 versus TNF‐α alone. (B) Cells were fixed with paraformaldehyde and incubated with primary antibody against ICAM‐1. Goat anti‐mouse FITC conjugated and DAPI were used for fluorescence detection of cells (green) and nuclei (blue) and images were captured at 10× magnifications. (a) asal (untreated) control; (b) TNF‐α; (c) MYO + TNF‐α.

The expression of the adhesion molecule Intercellular Adhesion Molecule‐1 (ICAM‐1) is upregulated in hypertrophic adipocytes during obesity and has been shown to play a pivotal role in monocyte adhesion (D. K. Brake et al. 2006). Therefore, we directly examined the effect of MYO on the regulation of ICAM‐1 gene and protein expression. As shown in Figure 3A, MYO significantly reduced ICAM‐1 gene and protein expression by approximately 60% and 30%, respectively. Immunocytochemical analyses confirmed the potential adhesion‐promoting role of ICAM‐1 by highlighting its localization at the periphery of adipocytes, particularly near the plasma membrane (as indicated in Figure 3B by the orange arrows), suggesting its mechanistic role in supporting monocyte adhesion.

**FIGURE 4:**
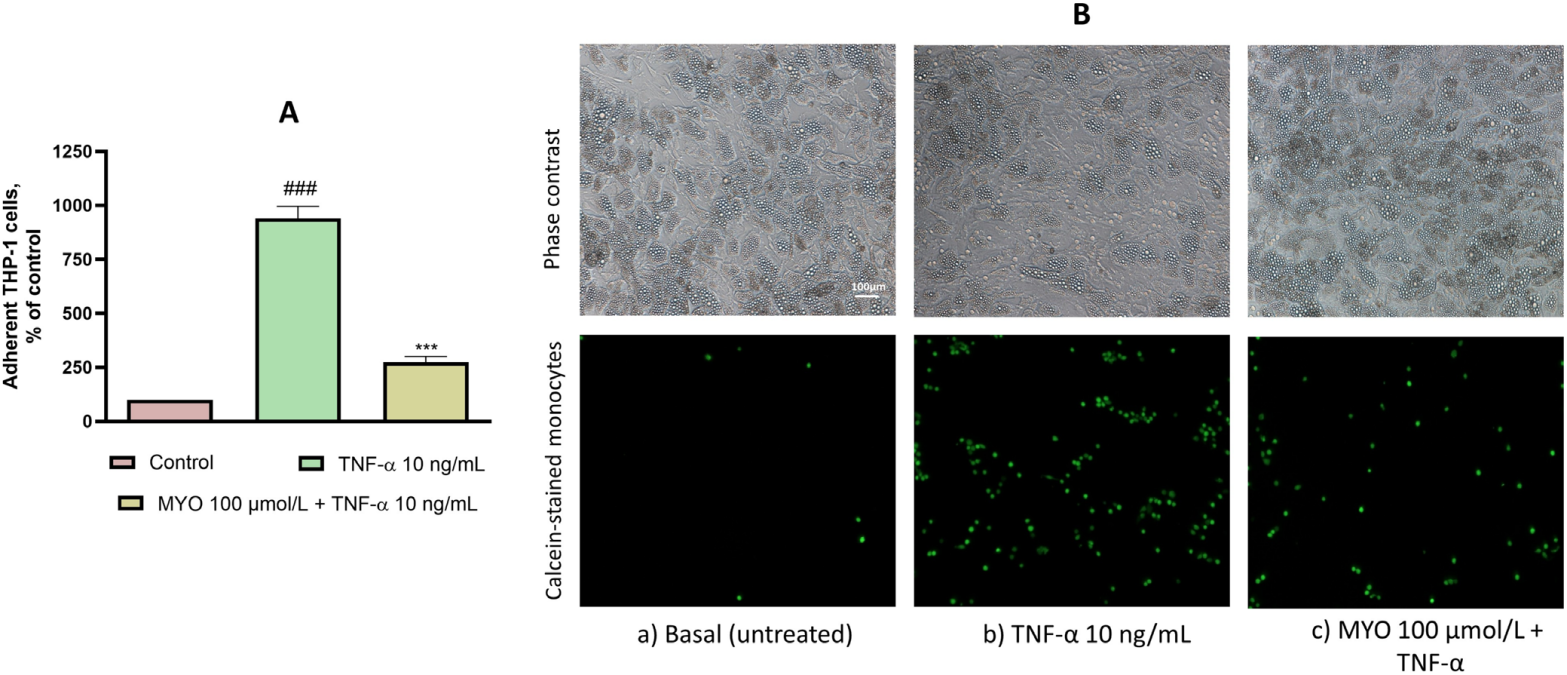
MYO reduces the adhesion of monocytes to inflamed adipocytes. Fluorescently labelled suspended THP‐1 (10^6^ cells/mL) were added to the SGBS monolayers. After 1 h, the non‐adherent cells were removed by washing three times, and the images of SGBS and adherent calcein‐labelled THP‐1 cells were visualised and recorded using a fluorescence microscope at 10× magnification. (A) Data (mean ± SD, *n* = 3) are expressed as % of adherent monocytes compared to control. ###*p* < 0.001 versus basal (untreated) control; ***p < 0.001 versus TNF‐α alone. (B) Representative images of the adhesion test.

To investigate the functional consequences of MYO‐mediated reduction in ICAM‐1 expression, we developed an adipocyte‐monocyte adhesion assay to evaluate monocyte adhesion. We observed that MYO exposure significantly reduced monocyte adhesion to inflamed adipocytes (Figure 4A,B), further functionally confirming the strong anti‐inflammatory effects of MYO in adipose cells. The role of ICAM‐1 was further elucidated by adding an anti‐ICAM‐1 antibody solution to TNF‐α‐stimulated cells during the adhesion assay, resulting in a significant reduction in monocyte adhesion. This finding suggests that ICAM‐1 plays a key role in mediating the adhesion of monocytes to inflamed adipocytes.

### 3.4 MYO Decreases the Cellular ROS and the Activation of NF‐κB in Inflamed Adipocytes

**FIGURE 5:**
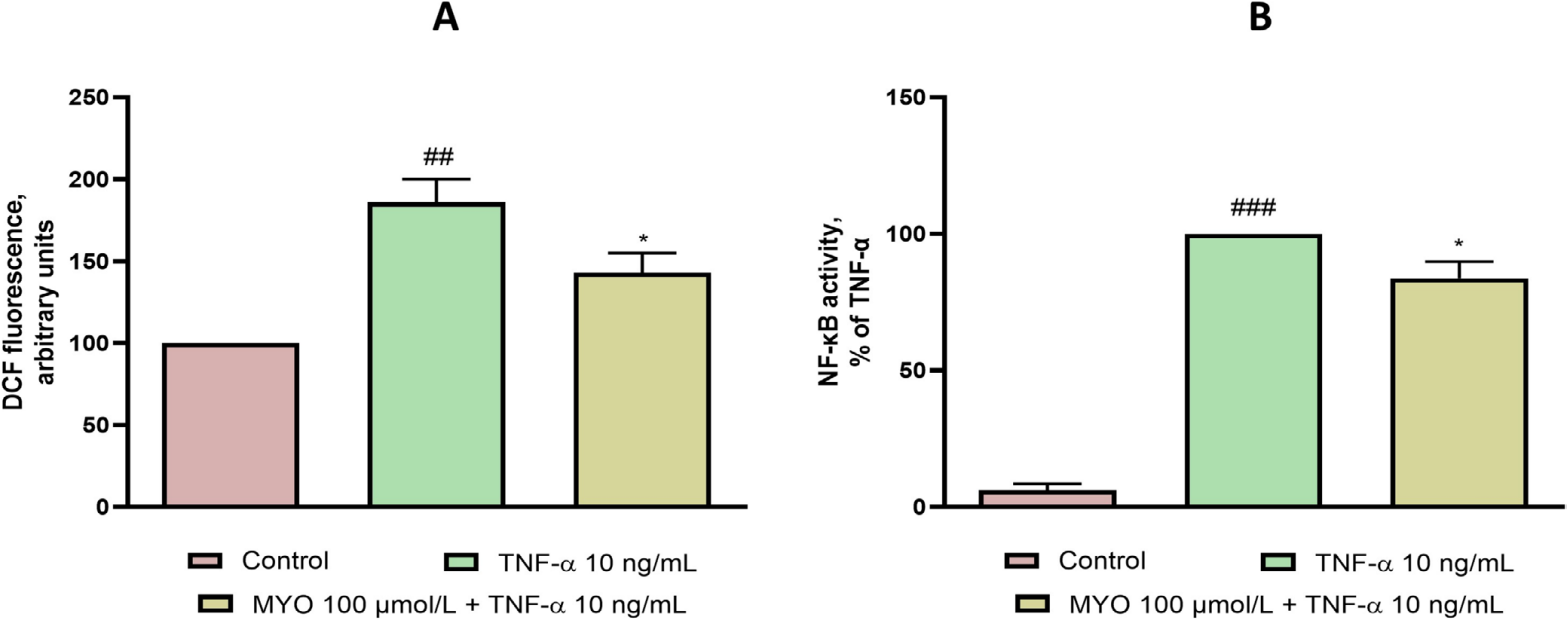
Effect of MYO on TNF‐α‐induced ROS generation and TNF‐α‐induced NF‐κB activation. (A) SGBS were treated with MYO for 4 h and then either treated with 10 ng/mL TNF‐α or left untreated for 1 h. ROS accumulation was determined by DCFH‐DA; fluorescence intensity was evaluated at 485 nm excitation and 535 nm emission. Data (means ± SD, *n* = 3) are expressed as % over control. ^##^ *p* < 0.001 versus basal (untreated) control; *p < 0.05 versus TNF‐α alone. (B) SGBS were treated with MYO for 4 h and then either treated with 10 ng/mL TNF‐α or left untreated for 1 h. Nuclear proteins were analyzed for NF‐κB p65 DNA‐binding activity by ELISA. Data (means ± SD, *n* = 3) are expressed as percent of TNF‐α. ^###^ *p* < 0.001 versus basal (untreated) control; *p < 0.05 versus TNF‐α alone.

NF‐κB is a key transcription factor involved in regulating adipocyte inflammation by promoting the expression of adhesion molecules, cytokines, and chemokines (Griffin 2022). Its activation is enhanced in proinflammatory, insulin‐resistant states driven by TNF‐α (Nieto‐Vazquez et al. 2008) and further mediated by excessive ROS production (Kuzmenko et al. 2016). Given MYO's insulin‐mimetic and antioxidant properties, we hypothesized that its anti‐inflammatory effects in human adipocytes might involve inhibition of ROS overproduction and NF‐κB activation. As shown in Figure 5, TNF‐α stimulation markedly increased both ROS levels (Figure 5A) and NF‐κB p65 DNA‐binding activity (Figure 5B). Pre‐treatment with MYO significantly attenuated these effects, reducing ROS production and NF‐κB activation. These findings suggest that MYO modulates proinflammatory pathway activation and gene expression in adipocytes (Yoon et al. 2012).

### 3.5 MYO Restore the Mitochondrial Content in Inflamed Adipocytes

Finally, considering that TNF‐α exposure in the context of obesity and insulin resistance is associated with altered mitochondrial dynamics through ROS‐ and NF‐κB‐mediated mechanisms (Chen et al. 2010; Mariappan et al. 2010; Valerio et al. 2006), we investigated the role of MYO in TNF‐α‐induced changes in mitochondrial content.

**FIGURE 6:**
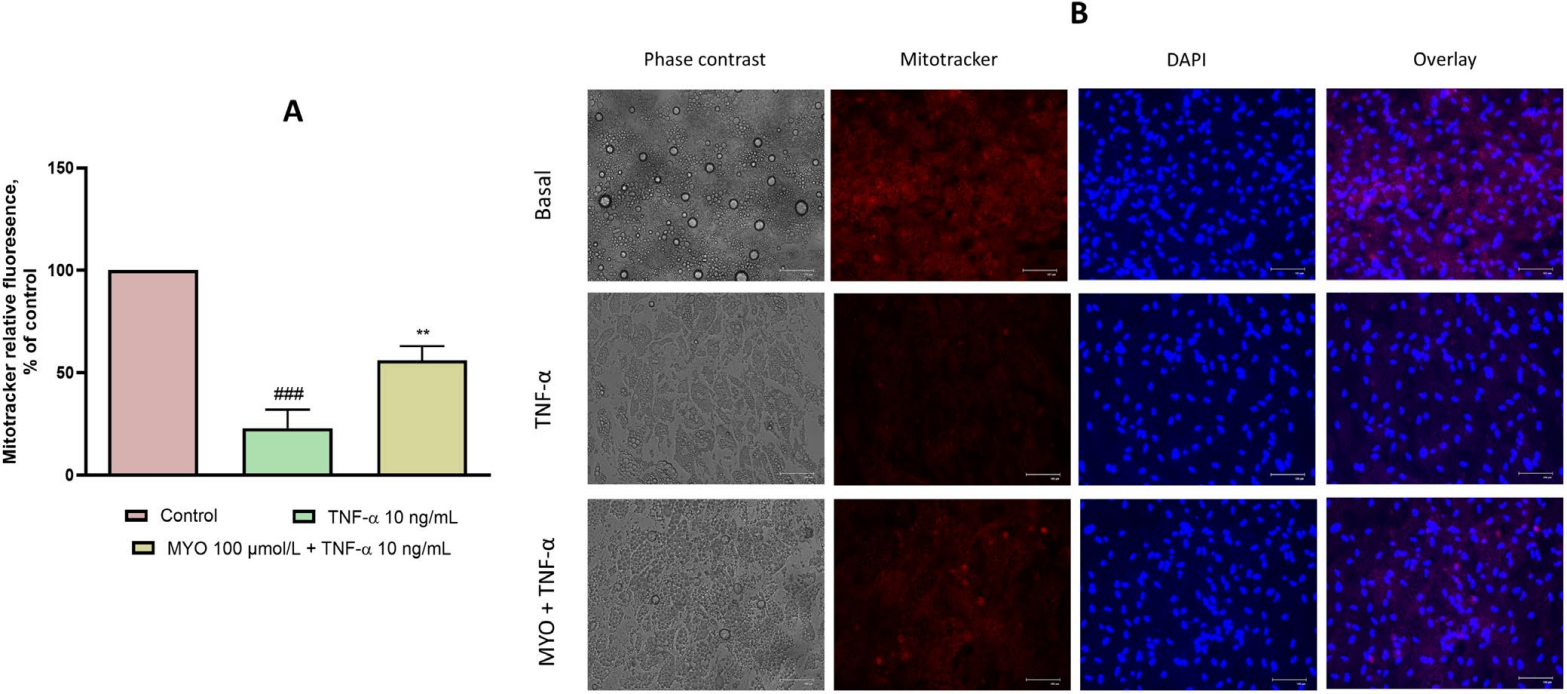
Effect of MYO on mitochondrial content. Mitochondrial content and mitochondrial biogenesis in various groups. SGBS were treated with 10 ng/mL of TNF‐α for 24 h in the presence or absence of 100 μmol/L MYO. (A) Relative fluorescence intensity of mitochondrial content: Fluorescence intensity was evaluated at 644 nm excitation and 665 nm emission. Data (means ± SD, *n* = 3) are expressed as % over control. ^###^ *p* < 0.001 versus basal (untreated) control; **p < 0.05 versus TNF‐α. (B) Fluorescent images of SGBS stained with mitotracker red (red). After mitotracker loading, cells were fixed with paraformaldehyde, nuclei stained by DAPI (blue) and images captured with a fluorescent microscope at 20× magnifications. Single‐channel images were overlayed and processed by microscope software. All scale bars are reported.

As shown in Figure 6, treatment with TNF‐α resulted in a significant reduction in mitochondrial content, which decreased 4.3‐fold compared to control (*p* < 0.001). However, in cells treated with MYO, mitochondrial density was significantly restored compared to TNF‐α treatment alone, showing a 2.2‐fold increase compared to the TNF‐α group (*p* < 0.05).

## 4 Discussion

This study provides a thorough molecular and functional analysis of the anti‐inflammatory properties of MYO in hypertrophic and inflamed adipose cells. Our results suggest that MYO effectively attenuates adipocyte activation triggered by dysmetabolic pro‐inflammatory stimuli, by reducing cytokine expression, monocyte migration, and adhesion to adipocytes, decreasing NF‐κB activation, and restoring mitochondrial content.

Under prolonged obesogenic conditions, enlarged adipose cells contribute to a persistent low‐grade inflammatory state that may accelerate the progression from early metabolic dysfunction to overt T2D and associated cardiovascular complications, particularly in individuals with central obesity (Shimobayashi et al. 2018). Both observational and experimental studies have reported increased infiltration of monocytes and macrophages in hypertrophic adipose tissue (Curat et al. 2006). This infiltration contributes to the local and systemic cytokine and lipid profile alteration and functionally sustains the development of the IR condition (Kurylowicz and Kozniewski 2020).

Therefore, the development of novel therapeutic approaches, including nutraceuticals or bioactive nutritional supplements, should aim to both counteract the pro‐inflammatory transition in adipose tissue and inhibit monocyte infiltration, thus intervening at multiple levels to alleviate the local and systemic metabolic dysfunction.

Over the past decade, INSs have gained growing clinical interest due to their involvement in various physiological processes, such as endocrine and metabolic functions, stress response, signal transduction following insulin and neurotransmitter triggering, as well as in calcium metabolism and cell proliferation (Bizzarri et al. 2016). INSs are hexahydroxycyclohexane alcohols that belong to the glucose family (Siddiqui et al. 2015). The two most important stereoisomers found in the human body are MYO and its derivative DCI (Bizzarri et al. 2016). Although both prokaryotic and eukaryotic cells may synthesize MYO, humans acquire it mainly through dietary sources in its free form, as inositol‐containing phospholipids, or as phytic acid (Bizzarri et al. 2016). Upon endogenous synthesis or external uptake, MYO is incorporated into cell membranes as phosphatidyl‐myo‐inositol, where it serves as the precursor for inositol triphosphate (IP3) and acts as a second messenger in various biological processes and signaling pathways, regulating the activities of hormones such as insulin, follicle‐stimulating hormone, and thyroid‐stimulating hormone (Bizzarri et al. 2016). It participates in the regulation of chromatin remodeling and gene expression (Odom et al. 2000; Shen et al. 2003) and facilitates mRNA export from the nucleus (York et al. 1999) besides being involved in the release of cytosolic calcium from intracellular calcium stores, such as the endoplasmic reticulum (Streb et al. 1983). Additionally, MYO plays a critical role in oocyte maturation and is reported to be involved in the physiological activities of testes, prostate, epididymis, seminal vesicles, and seminal fluid, which is one of the richest sources of inositols (Chiu et al. 2003).

The widespread role of INSs in the regulation of cell function suggests that an inositol deficiency could play a role in the development of various diseases. Inositol deficiency may result from several factors, including decreased dietary intake, increased catabolism and/or excretion, decreased biosynthesis and cellular uptake, and alterations in the gut microbiota (Holub 1986), so its supplementation may be of particular importance in certain pathologic conditions. For example, the conversion of MYO to DCI is severely impaired in patients with IR and T2D who have reduced plasma and tissue levels of DCI (Bevilacqua and Bizzarri 2018). Consistent with this, growing direct and indirect evidence demonstrates that supplementation with both MYO and DCI significantly improves the glycemic level in the absence of significant health risks (Caputo et al. 2020; DiNicolantonio and O'Keefe 2022).

Several mechanisms have been invoked to mechanistically explain such therapeutic potential. Undoubtedly, the intrinsic insulin‐mimetic activity (Sánchez‐Hidalgo et al. 2021) of MYO and DCI contributes to significantly support the reactivation of PI3K/Akt and favor insulin sensitivity in peripheral tissues (Cheng et al. 2019). Secondly, given the tight pathophysiological relationship between obesity and IR, MYO seems to indirectly contribute to rescue IR due to its ability to improve several anthropometric measures including weight and BMI (S. Arefhosseini, Roshanravan, Tutunchi, et al. 2023; Zarezadeh et al. 2022).

In this context, it should be considered that obese individuals have higher TNF‐α expression in adipose tissue (Quarta, Massaro, et al. 2022), which contributes to insulin signaling dysfunction by inhibiting PI3K/Akt pathway activation (de Alvaro et al. 2004) and promoting ROS production along with activation of pro‐inflammatory molecular switches. For this reason, we wanted to investigate whether and how MYO modulates TNF‐α‐induced pro‐inflammatory signaling, with particular emphasis on identifying molecular targets involved in monocyte/macrophage recruitment from adipose tissue. To achieve this, we utilized fully differentiated and hypertrophic SGBS cells as a cellular model of obesity, inducing insulin resistance and inflammation by exposing them to exogenous TNF‐α (Quarta et al. 2021). Among the thousands of genes upregulated in IR states exacerbated by TNF‐α, we chose to investigate the modulation of MCP‐1, IL‐6, and CXCL‐10 by MYO. In addition to the long‐known role of MCP‐1 in the recruitment of monocyte/macrophage to adipose tissue (Kanda et al. 2006), recent evidence suggests that IL‐6 also plays a dual role in dysfunctional adipose metabolism by regulating glucose utilization, lipolysis, oxidative metabolism, and energy expenditure while promoting adipose tissue inflammation (Han et al. 2020). In particular, IL‐6 from adipocytes has been shown to enhance macrophage infiltration, highlighting the importance of its cellular source in the expression of its activity (Han et al. 2020). Similarly, CXCL‐10 has been shown to play a critical role in the recruitment and adherence of leukocytes to endothelial cells, particularly in obese individuals (Moreno et al. 2022). Following chemokine‐mediated cell attraction, monocyte adhesion and infiltration are mediated by cell expression of the adhesion molecule ICAM‐1 (D. K. Brake et al. 2006; Jeong et al. 2015). Such a persistent inflammatory interaction forms a deleterious feedback loop that perpetuates local and systemic metabolic dysfunction. For this reason, the search for novel bioactive compounds that can modulate the expression of these chemokines and adhesion molecules is of great clinical importance.

We found that MYO, at concentrations equivalent to those achievable in vivo (Brown et al. 2009; R. S. Clements Jr. and Reynertson 1977), selectively downregulated TNF‐α‐induced gene expression of IL‐6, MCP‐1, and CXC‐L10 without signs of toxicity and independent of changes in lipid content. This was accompanied by a decrease in the corresponding protein release, which functionally resulted in a reduced ability to attract and maintain monocyte adhesion, partly due to reduced expression of the leukocyte adhesion molecule ICAM‐1. In an animal model of diet‐induced obesity, inflammation has been shown to upregulate the expression of ICAM‐1 in adipose tissue (D. K. Brake et al. 2006; Jeong et al. 2015). As far as we know, this is the first comprehensive demonstration of the direct and specific anti‐inflammatory effect of MYO in adipose cell models in terms of reducing adipokines and adhesion molecules. Our data corroborate those reported by Zhang et al. (2020) in an animal model of polycystic ovary syndrome and by Arefhosseini in obese patients (Arefhosseini, Roshanravan, Asghari, et al. 2023). Importantly, our results also complement those of Montt Guevara et al. and Monastra et al. who, using the same cell model, demonstrated the ability of MYO and DCI to activate cell browning (Montt Guevara et al. 2021) and insulin sensitization (Monastra et al. 2023). Thus, our study provides comprehensive evidence for the ability of inositols to improve the dysmetabolic and inflammatory features of adipose tissue under IR conditions.

To tentatively explore the underlying mechanisms of MYO action, we focused our efforts on testing the anti‐oxidant properties invoked for INSs (Rolnik et al. 2024) in relation to the activation of the well‐known redox‐sensitive transcription factor NF‐κB and alteration in mitochondrial content and function.

In obesity, the remodeling of adipose tissue is driven by a complex network of regulatory signals that ultimately converge on the activation of NF‐κB and JNK pathways (Lee and Lee 2014). This process is mediated, in part, by excessive production of ROS, which not only amplifies inflammatory signaling but also contributes to impaired mitochondrial functions. On the other hand, adequate mitochondrial functionality is now widely recognized as a key factor in maintaining metabolic health, with dysfunction playing a significant role in the development of obesity and insulin resistance (Lowell and Shulman 2005). Altered mitochondrial mass and structure have been observed in the adipocytes of *ob/ob* mice, and these changes were found normalized by the insulin‐sensitizing drug rosiglitazone (Wilson‐Fritch et al. 2004) and, more recently, by antioxidant treatments (Anusree et al. 2015). Moreover, the development of mitochondrial dysfunction in adipocytes has been identified as an early step in the pathogenesis of obesity‐associated IR (Guilherme et al. 2008). In support of this, Mariappan et al. demonstrated a causal role for NF‐κB–induced oxidative stress in promoting mitochondrial dysfunction in T2D (Mariappan et al. 2010). This oxidative imbalance renders adipocytes particularly susceptible to therapeutic interventions with antioxidant properties (Fu et al. 2025). Given that NF‐κB binding sites are present in the promoter regions of key pro‐inflammatory mediators, including MCP‐1 (Tsuchiya et al. 2006), ICAM‐1 (Xue et al. 2009), IL‐6 (Yoon et al. 2012), and CXCL‐10 (Yeruva et al. 2008), we investigated whether MYO, known for its antioxidant activity (Rolnik et al. 2024), could suppress NF‐κB activation and restore mitochondrial content.

To address this, we evaluated the effect of MYO on NF‐κB activity and mitochondrial content in adipocytes exposed to TNF‐α–induced oxidative stress. As expected, MYO significantly inhibited NF‐κB activation, in line with previous findings (El‐Hamoly et al. 2024).

Importantly, to our knowledge, this is the first study to demonstrate a direct effect of MYO on NF‐κB suppression in mature adipocytes. Moreover, and most notably, our data provide the first evidence that MYO not only attenuates oxidative stress and NF‐κB signaling, but also promotes a marked recovery of mitochondrial content under inflammatory conditions.

### 4.1 Limitations and Strengths

There are several limitations to consider when interpreting the results of this study. The primary limitation is that the data were obtained exclusively from human cell cultures. While these results provide valuable mechanistic insights into the effects of MYO, they are based on in vitro models and thus may not fully reflect the complex interactions occurring in vivo. Consequently, while these findings offer a starting point for understanding MYO's potential effects, they cannot be directly translated to clinical applications or patient outcomes. Only well‐designed clinical trials can provide the rigorous evidence necessary to confirm the relevance and applicability of these findings in human health contexts.

Additionally, while there is some preliminary in vivo evidence suggesting the potential benefits of MYO, there remains a significant gap in our understanding of how these effects manifest in actual human physiological conditions. This gap highlights the need for further investigation into the safety, efficacy, and potential therapeutic benefits of MYO in clinical settings. Additional research is necessary to further characterize the bioavailability, metabolism, and long‐term effects of MYO supplementation or its use in dietary interventions, as these factors can significantly impact its clinical applicability. Finally, while this study explores the potential anti‐inflammatory effects of MYO in response to various pro‐inflammatory stimuli, it mechanistically focuses on a single signaling pathway. However, the immune system is highly complex, and inflammation involves a network of interconnected signaling pathways and regulatory nodes. By examining only one pathway, this study offers a limited view of MYO's potential anti‐inflammatory activity. To achieve a more comprehensive understanding, future research should investigate MYO's effects on additional inflammatory pathways and mediators, as well as its broader immunomodulatory actions across multiple signaling nodes and networks. Despite these limitations, the study's validity is strengthened by the rigorous methodology employed. Gene expression was analyzed at the mRNA level, and these findings were further validated and functionally confirmed through protein expression analysis. This dual approach of examining both mRNA and protein levels provides stronger evidence for the proposed mechanisms and adds credibility to the conclusions drawn. These findings serve as a solid foundation for future research, providing a critical starting point for more in‐depth studies that will explore the full scope of MYO's biological effects and its potential for clinical application.

## 5 Conclusions

In conclusion, we have shown for the first time that, within a complex adipogenic environment, MYO effectively restores adipose function by reducing monocyte adhesion and inflammation in pro‐inflammatory activated adipocytes. While additional research is necessary, these findings indicate that MYO specifically alleviates the pro‐inflammatory and dysmetabolic conditions of hypertrophic adipocytes, highlighting its potential as a nutraceutical intervention to address obesity‐related complications and insulin resistance.

## Author Contributions

**Stefano Quarta:** conceptualization (equal), data curation (equal), formal analysis (lead), investigation (equal), methodology (lead), writing – original draft (equal), writing – review and editing (equal). **Nadia Calabriso:** methodology (equal), writing – review and editing (equal). **Maria Annunziata Carluccio:** methodology (equal), writing – review and editing (equal). **Tiziano Verri:** writing – review and editing (equal). **Marta Lo Rizzo:** investigation (equal). **Martin Wabitsch:** resources (equal). **Michele Maffia:** writing – review and editing (equal). **Fabrizio Damiano:** methodology (equal), writing – review and editing (equal). **Luisa Siculella:** writing – review and editing (equal). **Giuseppe Santarpino:** funding acquisition (equal), resources (equal). **Marika Massaro:** conceptualization (equal), data curation (equal), formal analysis (equal), methodology (equal), resources (equal), writing – original draft (lead), writing – review and editing (equal).

## Conflicts of Interest

The authors declare no conflicts of interest.

## Data Availability

Data and materials are available after contact with the corresponding author.

## References

[fsn370962-bib-0001] Anusree, S. S. , V. M. Nisha , A. Priyanka , and K. G. Raghu . 2015. “Insulin Resistance by TNF‐α Is Associated With Mitochondrial Dysfunction in 3T3‐L1 Adipocytes and Is Ameliorated by Punicic Acid, a PPARγ Agonist.” Molecular and Cellular Endocrinology 413: 120–128. 10.1016/j.mce.2015.06.018. 26116231

[fsn370962-bib-0002] Arefhosseini, S. , N. Roshanravan , S. Asghari , H. Tutunchi , and M. Ebrahimi‐Mameghani . 2023. “Expression of Inflammatory Genes, WBC‐Derived Inflammatory Biomarkers and Liver Function Indices: Effects of Myo‐Inositol Supplementation in Obese Patients With NAFLD.” Journal of Functional Foods 104: 105524. 10.1016/j.jff.2023.105524.

[fsn370962-bib-0003] Arefhosseini, S. , N. Roshanravan , H. Tutunchi , S. Rostami , M. Khoshbaten , and M. Ebrahimi‐Mameghani . 2023. “Myo‐Inositol Supplementation Improves Cardiometabolic Factors, Anthropometric Measures, and Liver Function in Obese Patients With Non‐Alcoholic Fatty Liver Disease.” Frontiers in Nutrition 10: 1092544. 10.3389/fnut.2023.1092544. 36824177 PMC9941177

[fsn370962-bib-0004] Bevilacqua, A. , and M. Bizzarri . 2018. “Inositols in Insulin Signaling and Glucose Metabolism.” International Journal of Endocrinology 2018: 1968450. 10.1155/2018/1968450. 30595691 PMC6286734

[fsn370962-bib-0005] Bizzarri, M. , A. Fuso , S. Dinicola , A. Cucina , and A. Bevilacqua . 2016. “Pharmacodynamics and Pharmacokinetics of Inositol(s) in Health and Disease.” Expert Opinion on Drug Metabolism & Toxicology 12, no. 10: 1181–1196. 10.1080/17425255.2016.1206887. 27351907

[fsn370962-bib-0006] Boutens, L. , G. J. Hooiveld , S. Dhingra , R. A. Cramer , M. G. Netea , and R. Stienstra . 2018. “Unique Metabolic Activation of Adipose Tissue Macrophages in Obesity Promotes Inflammatory Responses.” Diabetologia 61, no. 4: 942–953. 10.1007/s00125-017-4526-6. 29333574 PMC6448980

[fsn370962-bib-0007] Brake, D. K. , E. O. Smith , H. Mersmann , C. W. Smith , and R. L. Robker . 2006. “ICAM‐1 Expression in Adipose Tissue: Effects of Diet‐Induced Obesity in Mice.” American Journal of Physiology‐Cell Physiology 291, no. 6: C1232–C1239. 10.1152/ajpcell.00008.2006. 16807303

[fsn370962-bib-0008] Brown, L. D. , A. Cheung , J. E. Harwood , and F. C. Battaglia . 2009. “Inositol and Mannose Utilization Rates in Term and Late‐Preterm Infants Exceed Nutritional Intakes.” Journal of Nutrition 139, no. 9: 1648–1652. 10.3945/jn.109.109108. 19494026 PMC2728690

[fsn370962-bib-0009] Caputo, M. , E. Bona , I. Leone , et al. 2020. “Inositols and Metabolic Disorders: From Farm to Bedside.” Journal of Traditional and Complementary Medicine 10, no. 3: 252–259. 10.1016/j.jtcme.2020.03.005. 32670820 PMC7340869

[fsn370962-bib-0066] Chen, X. H. , Y. P. Zhao , M. Xue , et al. 2010. “TNF‐Alpha Induces Mitochondrial Dysfunction in 3T3‐L1 Adipocytes.” Molecular and Cellular Endocrinology 328, no. 1‐2: 63–69. 10.1016/j.mce.2010.07.005. 20667497

[fsn370962-bib-0010] Cheng, F. , L. Han , Y. Xiao , et al. 2019. “D‐Chiro‐Inositol Ameliorates High Fat Diet‐Induced Hepatic Steatosis and Insulin Resistance via PKCepsilon‐PI3K/AKT Pathway.” Journal of Agricultural and Food Chemistry 67, no. 21: 5957–5967. 10.1021/acs.jafc.9b01253. 31066268

[fsn370962-bib-0011] Chiu, T. T. , M. S. Rogers , C. Briton‐Jones , and C. Haines . 2003. “Effects of Myo‐Inositol on the In‐Vitro Maturation and Subsequent Development of Mouse Oocytes.” Human Reproduction 18, no. 2: 408–416. 10.1093/humrep/deg113. 12571181

[fsn370962-bib-0013] Clements, R. , and B. Darnell . 1980. “Myo‐Inositol Content of Common Foods: Development of a High‐Myo‐Inositol Diet.” American Journal of Clinical Nutrition 33: 1954–1967. 10.1093/ajcn/33.9.1954. 7416064

[fsn370962-bib-0012] Clements, R. S., Jr. , and R. Reynertson . 1977. “Myoinositol Metabolism in Diabetes Mellitus. Effect of Insulin Treatment.” Diabetes 26, no. 3: 215–221. 10.2337/diab.26.3.215. 838172

[fsn370962-bib-0014] Curat, C. A. , V. Wegner , C. Sengenes , et al. 2006. “Macrophages in Human Visceral Adipose Tissue: Increased Accumulation in Obesity and a Source of Resistin and Visfatin.” Diabetologia 49, no. 4: 744–747. 10.1007/s00125-006-0173-z. 16496121

[fsn370962-bib-0015] de Alvaro, C. , T. Teruel , R. Hernandez , and M. Lorenzo . 2004. “Tumor Necrosis Factor α Produces Insulin Resistance in Skeletal Muscle by Activation of Inhibitor κB Kinase in a p38 MAPK‐Dependent Manner.” Journal of Biological Chemistry 279, no. 17: 17070–17078. 10.1074/jbc.M312021200. 14764603

[fsn370962-bib-0016] DiNicolantonio, J. J. , and J. H. O'Keefe . 2022. “Myo‐Inositol for Insulin Resistance, Metabolic Syndrome, Polycystic Ovary Syndrome and Gestational Diabetes.” Open Heart 9, no. 1: e001989. 10.1136/openhrt-2022-001989. 35236761 PMC8896029

[fsn370962-bib-0017] El‐Hamoly, T. , G. Shafey , and M. El‐Sheikh . 2024. “Modulation of FOXO1/NF‐κB Signaling via Inositol Alleviates the Radiation‐Induced Brain Injury.” Egyptian Journal of Radiation Sciences and Applications 3: 69–77. 10.21608/ejrsa.2024.305083.1172.

[fsn370962-bib-0018] Fu, M. , K.‐S. Yoon , J. Ha , I. Kang , and W. Choe . 2025. “Crosstalk Between Antioxidants and Adipogenesis: Mechanistic Pathways and Their Roles in Metabolic Health.” Antioxidants 14, no. 2: 203. 40002389 10.3390/antiox14020203 PMC11852089

[fsn370962-bib-0019] Fujishiro, M. , Y. Gotoh , H. Katagiri , et al. 2003. “Three Mitogen‐Activated Protein Kinases Inhibit Insulin Signaling by Different Mechanisms in 3T3‐L1 Adipocytes.” Molecular Endocrinology 17, no. 3: 487–497. 10.1210/me.2002-0131. 12554784

[fsn370962-bib-0020] Ghanbari, M. , S. Momen Maragheh , A. Aghazadeh , et al. 2021. “Interleukin‐1 in Obesity‐Related Low‐Grade Inflammation: From Molecular Mechanisms to Therapeutic Strategies.” International Immunopharmacology 96: 107765. 10.1016/j.intimp.2021.107765. 34015596

[fsn370962-bib-0021] Goldfine, A. B. , and S. E. Shoelson . 2017. “Therapeutic Approaches Targeting Inflammation for Diabetes and Associated Cardiovascular Risk.” Journal of Clinical Investigation 127, no. 1: 83–93. 10.1172/JCI88884. 28045401 PMC5199685

[fsn370962-bib-0022] Griffin, M. J. 2022. “On the Immunometabolic Role of NF‐κB in Adipocytes.” Immunometabolism 4, no. 1: e220003. 10.20900/immunometab20220003. 35251704 PMC8893669

[fsn370962-bib-0023] Guilherme, A. , J. V. Virbasius , V. Puri , and M. P. Czech . 2008. “Adipocyte Dysfunctions Linking Obesity to Insulin Resistance and Type 2 Diabetes.” Nature Reviews. Molecular Cell Biology 9, no. 5: 367–377. 10.1038/nrm2391. 18401346 PMC2886982

[fsn370962-bib-0024] Han, M. S. , A. White , R. J. Perry , et al. 2020. “Regulation of Adipose Tissue Inflammation by Interleukin 6.” Proceedings of the National Academy of Sciences of the United States of America 117, no. 6: 2751–2760. 10.1073/pnas.1920004117. 31980524 PMC7022151

[fsn370962-bib-0025] Hersoug, L.‐G. , P. Møller , and S. Loft . 2018. “Role of Microbiota‐Derived Lipopolysaccharide in Adipose Tissue Inflammation, Adipocyte Size and Pyroptosis During Obesity.” Nutrition Research Reviews 31, no. 2: 153–163. 10.1017/S0954422417000269. 29362018

[fsn370962-bib-0026] Holub, B. J. 1986. “Metabolism and Function of Myo‐Inositol and Inositol Phospholipids.” Annual Review of Nutrition 6: 563–597. 10.1146/annurev.nu.06.070186.003023. 2425833

[fsn370962-bib-0027] Hotamisligil, G. S. , P. Arner , J. F. Caro , R. L. Atkinson , and B. M. Spiegelman . 1995. “Increased Adipose Tissue Expression of Tumor Necrosis Factor‐Alpha in Human Obesity and Insulin Resistance.” Journal of Clinical Investigation 95, no. 5: 2409–2415. 10.1172/JCI117936. 7738205 PMC295872

[fsn370962-bib-0028] Ishizuka, K. , I. Usui , Y. Kanatani , et al. 2007. “Chronic Tumor Necrosis Factor‐α Treatment Causes Insulin Resistance via Insulin Receptor Substrate‐1 Serine Phosphorylation and Suppressor of Cytokine Signaling‐3 Induction in 3T3‐L1 Adipocytes.” Endocrinology 148, no. 6: 2994–3003. 10.1210/en.2006-1702. 17379643

[fsn370962-bib-0029] Jeong, J. H. , Y. R. Lee , H. G. Park , and W. L. Lee . 2015. “The Effects of Either Resveratrol or Exercise on Macrophage Infiltration and Switching From M1 to M2 in High Fat Diet Mice.” Journal of Exercise Nutrition & Biochemistry 19, no. 2: 65–72. 10.5717/jenb.2015.15060203. 26244124 PMC4523807

[fsn370962-bib-0030] Kanda, H. , S. Tateya , Y. Tamori , et al. 2006. “MCP‐1 Contributes to Macrophage Infiltration Into Adipose Tissue, Insulin Resistance, and Hepatic Steatosis in Obesity.” Journal of Clinical Investigation 116, no. 6: 1494–1505. 10.1172/jci26498. 16691291 PMC1459069

[fsn370962-bib-0031] Kurylowicz, A. , and K. Kozniewski . 2020. “Anti‐Inflammatory Strategies Targeting Metaflammation in Type 2 Diabetes.” Molecules 25, no. 9: 2224. 10.3390/molecules25092224. 32397353 PMC7249034

[fsn370962-bib-0032] Kuzmenko, D. I. , S. N. Udintsev , T. K. Klimentyeva , and V. Y. Serebrov . 2016. “Oxidative Stress in Adipose Tissue as a Primary Link in Pathogenesis of Insulin Resistance.” Biochemistry (Moscow), Supplement Series B: Biomedical Chemistry 10, no. 3: 212–219. 10.1134/S1990750816030100. 26973182

[fsn370962-bib-0033] Lee, B. C. , and J. Lee . 2014. “Cellular and Molecular Players in Adipose Tissue Inflammation in the Development of Obesity‐Induced Insulin Resistance.” Biochimica et Biophysica Acta 1842, no. 3: 446–462. 10.1016/j.bbadis.2013.05.017. 23707515 PMC3800253

[fsn370962-bib-0034] Lowell, B. B. , and G. I. Shulman . 2005. “Mitochondrial Dysfunction and Type 2 Diabetes.” Science 307, no. 5708: 384–387. 10.1126/science.1104343. 15662004

[fsn370962-bib-0035] Mariappan, N. , C. M. Elks , S. Sriramula , et al. 2010. “NF‐kappaB‐Induced Oxidative Stress Contributes to Mitochondrial and Cardiac Dysfunction in Type II Diabetes.” Cardiovascular Research 85, no. 3: 473–483. 10.1093/cvr/cvp305. 19729361 PMC2860708

[fsn370962-bib-0036] Massaro, M. , G. Basta , G. Lazzerini , et al. 2002. “Quenching of Intracellular ROS Generation as a Mechanism for Oleate‐Induced Reduction of Endothelial Activation and Early Atherogenesis.” Thrombosis and Haemostasis 88, no. 2: 335–344. 12195709

[fsn370962-bib-0037] Massaro, M. , E. Scoditti , M. Pellegrino , et al. 2016. “Therapeutic Potential of the Dual Peroxisome Proliferator Activated Receptor (PPAR)alpha/Gamma Agonist Aleglitazar in Attenuating TNF‐Alpha‐Mediated Inflammation and Insulin Resistance in Human Adipocytes.” Pharmacological Research 107: 125–136. 10.1016/j.phrs.2016.02.027. 26976796

[fsn370962-bib-0038] Monastra, G. , R. Gambioli , V. Unfer , G. Forte , E. Maymo‐Masip , and R. Comitato . 2023. “D‐Chiro‐Inositol and Myo‐Inositol Induce WAT/BAT Trans‐Differentiation in Two Different Human Adipocyte Models (SGBS and LiSa‐2).” International Journal of Molecular Sciences 24, no. 8: 7421. 10.3390/ijms24087421. 37108582 PMC10139407

[fsn370962-bib-0039] Montt Guevara, M. , M. Finiguerra , I. Marzi , et al. 2021. “D‐Chiro‐Inositol Regulates Insulin Signaling in Human Adipocytes.” Frontiers in Endocrinology 12: 660815. 10.3389/fendo.2021.660815. 33859622 PMC8042392

[fsn370962-bib-0040] Moreno, B. , L. Hueso , R. Ortega , et al. 2022. “Association of Chemokines IP‐10/CXCL10 and I‐TAC/CXCL11 With Insulin Resistance and Enhance Leukocyte Endothelial Arrest in Obesity.” Microvascular Research 139: 104254. 10.1016/j.mvr.2021.104254. 34534571

[fsn370962-bib-0041] Nieto‐Vazquez, I. , S. Fernández‐Veledo , D. K. Krämer , R. Vila‐Bedmar , L. Garcia‐Guerra , and M. Lorenzo . 2008. “Insulin Resistance Associated to Obesity: The Link TNF‐Alpha.” Archives of Physiology and Biochemistry 114, no. 3: 183–194. 10.1080/13813450802181047. 18629684

[fsn370962-bib-0042] Odom, A. R. , A. Stahlberg , S. R. Wente , and J. D. York . 2000. “A Role for Nuclear Inositol 1,4,5‐Trisphosphate Kinase in Transcriptional Control.” Science 287, no. 5460: 2026–2029. 10.1126/science.287.5460.2026. 10720331

[fsn370962-bib-0043] Pani, A. , R. Giossi , D. Menichelli , et al. 2020. “Inositol and Non‐Alcoholic Fatty Liver Disease: A Systematic Review on Deficiencies and Supplementation.” Nutrients 12, no. 11: 3379. 33153126 10.3390/nu12113379 PMC7694137

[fsn370962-bib-0044] Quarta, S. , M. Massaro , M. A. Carluccio , et al. 2022. “An Exploratory Critical Review on TNF‐Alpha as a Potential Inflammatory Biomarker Responsive to Dietary Intervention With Bioactive Foods and Derived Products.” Food 11, no. 16: 2524. 10.3390/foods11162524. PMC9407274 36010524

[fsn370962-bib-0045] Quarta, S. , G. Santarpino , M. A. Carluccio , et al. 2022. “Analysis of the Anti‐Inflammatory and Anti‐Osteoarthritic Potential of Flonat Fast®, a Combination of *Harpagophytum procumbens* DC. ex Meisn., *Boswellia serrata* Roxb., *Curcuma longa* L., Bromelain and Escin (*Aesculus hippocastanum*), Evaluated in In Vitro Models of Inflammation Relevant to Osteoarthritis.” Pharmaceuticals 15, no. 10: 1263. 10.3390/ph15101263. 36297375 PMC9609228

[fsn370962-bib-0046] Quarta, S. , E. Scoditti , M. A. Carluccio , et al. 2021. “Coffee Bioactive N‐Methylpyridinium Attenuates Tumor Necrosis Factor (TNF)‐Alpha‐Mediated Insulin Resistance and Inflammation in Human Adipocytes.” Biomolecules 11, no. 10: 1545. 10.3390/biom11101545. 34680177 PMC8534185

[fsn370962-bib-0047] Rolnik, A. , B. Olas , J. Szablińska‐Piernik , L. B. Lahuta , L. Gromadziński , and M. S. Majewski . 2024. “Antioxidant and Anticoagulant Properties of Myo‐Inositol Determined in an Ex Vivo Studies and Gas Chromatography Analysis.” Scientific Reports 14, no. 1: 25633. 10.1038/s41598-024-76527-2. 39465311 PMC11514185

[fsn370962-bib-0048] Sánchez‐Hidalgo, M. , A. J. León‐González , M. Gálvez‐Peralta , N. H. González‐Mauraza , and C. Martin‐Cordero . 2021. “D‐Pinitol: A Cyclitol With Versatile Biological and Pharmacological Activities.” Phytochemistry Reviews 20, no. 1: 211–224. 10.1007/s11101-020-09677-6.

[fsn370962-bib-0049] Sethi, J. K. , and G. S. Hotamisligil . 2021. “Metabolic Messengers: Tumour Necrosis Factor.” Nature Metabolism 3, no. 10: 1302–1312. 10.1038/s42255-021-00470-z. 34650277

[fsn370962-bib-0050] Shen, X. , H. Xiao , R. Ranallo , W.‐H. Wu , and C. Wu . 2003. “Modulation of ATP‐Dependent Chromatin‐Remodeling Complexes by Inositol Polyphosphates.” Science 299, no. 5603: 112–114. 10.1126/science.1078068. 12434013

[fsn370962-bib-0051] Shimobayashi, M. , V. Albert , B. Woelnerhanssen , et al. 2018. “Insulin Resistance Causes Inflammation in Adipose Tissue.” Journal of Clinical Investigation 128, no. 4: 1538–1550. 10.1172/JCI96139. 29528335 PMC5873875

[fsn370962-bib-0052] Siddiqui, N. , V. Singh , M. M. Deshmukh , and R. Gurunath . 2015. “Structures, Stability and Hydrogen Bonding in Inositol Conformers.” Physical Chemistry Chemical Physics 17, no. 28: 18514–18523. 10.1039/c5cp02690c. 26108975

[fsn370962-bib-0053] Streb, H. , R. F. Irvine , M. J. Berridge , and I. Schulz . 1983. “Release of Ca^2+^ From a Nonmitochondrial Intracellular Store in Pancreatic Acinar Cells by Inositol‐1,4,5‐Trisphosphate.” Nature 306, no. 5938: 67–69. 10.1038/306067a0. 6605482

[fsn370962-bib-0054] Tsuchiya, K. , T. Yoshimoto , Y. Hirono , T. Tateno , T. Sugiyama , and Y. Hirata . 2006. “Angiotensin II Induces Monocyte Chemoattractant Protein‐1 Expression via a Nuclear Factor‐κB‐Dependent Pathway in Rat Preadipocytes.” American Journal of Physiology. Endocrinology and Metabolism 291, no. 4: E771–E778. 10.1152/ajpendo.00560.2005. 16705055

[fsn370962-bib-0067] Valerio, A. , A. Cardile , V. Cozzi , et al. 2006. “TNF‐Alpha Downregulates eNOS Expression and Mitochondrial Biogenesis in Fat and Muscle of Obese Rodents.” Journal of Clinical Investigation 116, no. 10: 2791–2798. 10.1172/jci28570. 16981010 PMC1564431

[fsn370962-bib-0055] Wabitsch, M. , R. E. Brenner , I. Melzner , et al. 2001. “Characterization of a Human Preadipocyte Cell Strain With High Capacity for Adipose Differentiation.” International Journal of Obesity 25, no. 1: 8–15. 10.1038/sj.ijo.0801520. 11244452

[fsn370962-bib-0056] Wilson‐Fritch, L. , S. Nicoloro , M. Chouinard , et al. 2004. “Mitochondrial Remodeling in Adipose Tissue Associated With Obesity and Treatment With Rosiglitazone.” Journal of Clinical Investigation 114, no. 9: 1281–1289. 10.1172/JCI21752. 15520860 PMC524228

[fsn370962-bib-0057] Wu, H. , and C. M. Ballantyne . 2020. “Metabolic Inflammation and Insulin Resistance in Obesity.” Circulation Research 126, no. 11: 1549–1564. 10.1161/CIRCRESAHA.119.315896. 32437299 PMC7250139

[fsn370962-bib-0058] Xue, J. , P. B. Thippegowda , G. Hu , et al. 2009. “NF‐KappaB Regulates Thrombin‐Induced ICAM‐1 Gene Expression in Cooperation With NFAT by Binding to the Intronic NF‐KappaB Site in the ICAM‐1 Gene.” Physiological Genomics 38, no. 1: 42–53. 10.1152/physiolgenomics.00012.2009. 19351910 PMC2696150

[fsn370962-bib-0059] Yeruva, S. , G. Ramadori , and D. Raddatz . 2008. “NF‐KappaB‐Dependent Synergistic Regulation of CXCL10 Gene Expression by IL‐1beta and IFN‐Gamma in Human Intestinal Epithelial Cell Lines.” International Journal of Colorectal Disease 23, no. 3: 305–317. 10.1007/s00384-007-0396-6. 18046562 PMC2225996

[fsn370962-bib-0060] Yoon, S. , S. U. Woo , J. H. Kang , et al. 2012. “NF‐κB and STAT3 Cooperatively Induce IL6 in Starved Cancer Cells.” Oncogene 31, no. 29: 3467–3481. 10.1038/onc.2011.517. 22105366

[fsn370962-bib-0061] York, J. D. , A. R. Odom , R. Murphy , E. B. Ives , and S. R. Wente . 1999. “A Phospholipase C‐Dependent Inositol Polyphosphate Kinase Pathway Required for Efficient Messenger RNA Export.” Science 285, no. 5424: 96–100. 10.1126/science.285.5424.96. 10390371

[fsn370962-bib-0062] Zarezadeh, M. , A. Dehghani , A. H. Faghfouri , et al. 2022. “Inositol Supplementation and Body Mass Index: A Systematic Review and Meta‐Analysis of Randomized Clinical Trials.” Obesity Science & Practice 8, no. 3: 387–397. 10.1002/osp4.569. 35664247 PMC9159559

[fsn370962-bib-0063] Zatterale, F. , M. Longo , J. Naderi , et al. 2019. “Chronic Adipose Tissue Inflammation Linking Obesity to Insulin Resistance and Type 2 Diabetes.” Frontiers in Physiology 10: 1607. 10.3389/fphys.2019.01607. 32063863 PMC7000657

[fsn370962-bib-0064] Zhang, Y. , C. Li , W. Zhang , X. Zheng , and X. Chen . 2020. “Decreased Insulin Resistance by Myo‐Inositol Is Associated With Suppressed Interleukin 6/Phospho‐STAT3 Signaling in a Rat Polycystic Ovary Syndrome Model.” Journal of Medicinal Food 23, no. 4: 375–387. 10.1089/jmf.2019.4580. 32045334

[fsn370962-bib-0065] Zhou, Y. , H. Li , and N. Xia . 2021. “The Interplay Between Adipose Tissue and Vasculature: Role of Oxidative Stress in Obesity.” Frontiers in Cardiovascular Medicine 8: 650214. 10.3389/fcvm.2021.650214. 33748199 PMC7969519

